# Aseptic Dressing of Penetrating and Punctured Wounds

**Published:** 1887-09

**Authors:** O. Grothan

**Affiliations:** Scotia, Nebraska


### ASEPTIC DRESSING OF PENETRATING AND PUNCTURED WOUNDS.

BY O. GROTHAN, M. D., SCOTIA, NEBRASKA.

The treatment of this kind of injuries, including gun-shot wounds, has been heretofore, and I find it is yet, to an alarming extent, so erroneously pursued, that a few remarks on this subject will, I hope, prove of interest to the readers of the *Gazette*.

It is not my intention or claim to advance any new ideas or elaborate theories, but shall confine myself to pathological facts, from actual observation, in a clinical point of view.

Having seen the many evil effects—such as continued and exhausting suppuration, the extravasation of pus into the cellular planes, thus producing abscesses and exposing the patient to the liability of septicæmia and pyæmia—of gun-shot wounds especially, treated according to the old method; it occurred to me that something should be accomplished in the treatment to prevent the dangerous consequences which follow the probing for bullets, and the drawing of handkerchiefs through the opening made by projectiles.

In searching the authorities on this subject I find nothing satisfactory, yet they all speak of hermetically sealing, as it were, compound fractures for the purpose of converting them into simple ones. The good results of this practice no one will dispute; and why, then, should not this be a guide to the treatment of punctured and penetrating wounds, where it is most assuredly as essential to get healing without suppuration? Let me say a few words of the pathological facts in support of this mode of treatment. Take a simple fracture with considerable laceration of tissue, and the extravasation of blood between the torn parts or the thrombus formed in the end of a large blood-vessel after ligation. Is this not foreign material as much as shreds of tissue after the infliction of a slightly lacerated punctured or penetrating wound, such as you find made by a bullet? Why, then, should we not hope for the same result in either case? And that we may is what I propose to show in the subsequent clinical report.

The pathological changes are the same, provided the treatment is so directed as to give these injuries, especially of the soft parts, excluding the peritoneal, pericardial and cranial cavities, the same chance for repair as simple fractures.

In the first place there must be a reorganization or reabsorption of the material occupying the space between the lacerated walls of the wound, as healing by first intention is out of the question, due to non-coaptation.

This material primarily consists of red blood corpuscles, leucocytes, serum, fine filaments of coagulated fibrin, and shreds of tissue. The changes which this substance must undergo is, in brief: First, the red corpuscles disappear by disintegration and absorption, the white cells increasing; second, liquification of the coagulated fibrin and shreds of tissue gradually taking place as the cell infiltration proceeds, serving as an agglutinating, intercellular substance capable of protoplastic change, by being taken up as nourishment for these cells. This change goes on in all wounds not disturbed by continued cleansing and washing, but at this stage the termination is determined. Suppose that by injection, insertion of probe, instruments, or finger, or admission of air, micro-coci, bacilli, or ferments, or whatever you may choose to call it, is admitted, these cells, through a metamorphic force, are converted into pus-cells and suppuration is fully established. But, on the other hand, if the wound is excluded from the air and ferments (I prefer this term, as no one as yet has established the exact nature of the substance producing this change), the pathological phenomenon is altogether different. This plastic material, which has undergone friable disintegration, assuming a cicatricial condition, the intercellular substance becomes fibrous, the cells assume a spindle shape, and are converted into connective tissue cells, and by the extension of shoots from the blood vessels from the adjacent parts, the repair is complete, in that an organized fibrous tissue is formed, gradually becoming dense from the disappearance of the blood vessels in part, which at first were very abundant. My method of treatment of this class of wounds is based upon the above facts, and, therefore, consists in, as soon as possible, excluding all source of fermentative infection by applying a dressing which is best adapted to accomplish this end. Many dressings may be suggested, but the one which has served best in my hands is iodoform in sufficient quantity to cover the wound, and, over this, dry absorbent cotton, covered with oil silk, kept in place by a well-applied bandage, and left in position as long as deemed advisable, usually from four to ten days, according to nature of wound—provided no unfavorable symptoms arise which would suggest the occurrence of irritation, excessive inflammation, or suppuration. In regard to the utility of probing a wound with the finger or instrument, I see none. Suppose you should find a bullet, or ascertain the depth of a punctured wound, what is accomplished except the establishment of infallible suppuration? A lead bullet in any part of the body will become encysted. Unless pressing on vital parts, such as brain or spinal cord, it is better not to interfere; and there are cases on record where recovery has ensued with a bullet in the cranial cavity. As to other foreign bodies, as shreds of clothing carried in by the projectile, they, as a rule, lodge immediately under the integument, and if they remain in the wound for some time nothing is lost, as eventually suppuration is only established superficially to a foreign body. The only cases where there is a question as to the propriety of this mode of treatment is where the intestinal viscera has been perforated, with extravasation of fecal matter, or severe splintering of bone, and laceration of large blood vessels has taken place. In the first an incision and coaptation of the intestine by cat-gut suture is probably to be advised. In the other, free incision is undoubtedly advisable, and treated as an open wound, but owing to the density and elasticity of the walls of the blood vessels, the least complication is not of frequent occurrence.

As to drawing handkerchiefs through wounds of this kind, it is hazardous to the utmost, and it is my full belief that more harm than good has been done in this way.

The only thing I can see in this old method of treatment is to impress your patient and his friends that you are doing something, while in reality helping him to eternity, or increasing the liability to be a cripple for life.

In regard to cutting out bullets, it is much better to leave them in their places of lodgment, even under the integument, until the wound has healed, and then extirpate it, than to remove it at once.

I will here state three cases which have been treated by myself within the last two months:

Case No. 1. Mr. S., age 74, on the 16th day of August, fell from a wagon heavily loaded, which ran over his left arm, producing a compound fracture of the lower third of humerus, with two openings. Owing to his profound intoxication, anæthesia was not advisable, and, being very muscular, the replacement was deferred to the next morning, but a dressing of iodoform applied. The next day the bandages were removed, but care taken so that the cotton, which was spread over a very small space, was not disturbed. The arm was then set, more cotton applied, with usual splints, and dressing not removed for twenty-one days, when a slight crust, easily removed by hot water, indicated the places of opening. The patient has now made a complete recovery, without further treatment.

Case No. 2. A child, 7 years old, on the 24th of August, fell from a chair, piercing its neck, a blade of a pair of scissors entering at the lower part of the superior carotid triangle, to the depth of two inches. I was called at once, and found the child bleeding quite profusely. The wound was dressed as the aforesaid plan is described, and as tight a compress was applied as the situation would allow. The bleeding ceased, and, after five days, the dressing removed, but the wound was not exposed. It seemed to be doing well. After four more days the entire dressing was removed, and the wound found to be completely healed.

Case No. 3. Mr. C., age about 30. On the 11th of September I was called in haste to the patient, living about five miles from town. On arrival I found him suffering from a gun-shot wound, the ball entering at the upper part of the thigh, internal, but immediately beside the femoral artery, inflicted by a charge of No. 8 shot, at about twenty inches from the muzzle of the gun. Bleeding had mostly ceased. On inquiry I found that the charge had first penetrated another man's coat and vest, glancing on a heavy leather pocket-book. I went in search of the wadding, but could not find any. Thinking it more than likely the wadding was retained in the clothing of the first man, I at once applied an aseptic dressing, iodoform, cotton, and oil silk, and covered by roller bandage. On the fifth day I saw the patient again, but finding no indication of febrile disturbance or excessive inflammation, the wound was left until the ninth day, when I removed the dressing, and found the wound to be crusted over. On removing the scab, a very small amount of pus exuded from the wound. After cleansing thoroughly, iodoform was again applied, with cotton saturated by a solution of borate of glycerine and sweet oil. The patient was then directed to dress the wound once daily, using the last-mentioned dressing. It suppurated slightly for four days, after which a scab formed and recovery was completed in a few days. The cotton becoming saturated with blood, seemed to be one of the principal factors in excluding air and septic germs from the wound.—*Southwestern Medical Gazette.*

